# Adhesion Molecules Involved in Stem Cell Niche Retention During Normal Haematopoiesis and in Acute Myeloid Leukaemia

**DOI:** 10.3389/fimmu.2021.756231

**Published:** 2021-11-12

**Authors:** Julien M. P. Grenier, Céline Testut, Cyril Fauriat, Stéphane J. C. Mancini, Michel Aurrand-Lions

**Affiliations:** Aix-Marseille Université, Centre National de la Recherche Scientifique (CNRS), Institut National de la Santé et de la Recherche Médicale (INSERM), Institut Paoli Calmettes, Centre de Recherche en Cancérologie de Marseille (CRCM), Equipe Labellisée Ligue Nationale Contre le Cancer 2020, Marseille, France

**Keywords:** adhesion, haematopoietic stem cell, leukemic stem cell, haematopoiesis, bone marrow, acute myeloid leukaemia

## Abstract

In the bone marrow (BM) of adult mammals, haematopoietic stem cells (HSCs) are retained in micro-anatomical structures by adhesion molecules that regulate HSC quiescence, proliferation and commitment. During decades, researchers have used engraftment to study the function of adhesion molecules in HSC’s homeostasis regulation. Since the 90’s, progress in genetically engineered mouse models has allowed a better understanding of adhesion molecules involved in HSCs regulation by BM niches and raised questions about the role of adhesion mechanisms in conferring drug resistance to cancer cells nested in the BM. This has been especially studied in acute myeloid leukaemia (AML) which was the first disease in which the concept of cancer stem cell (CSC) or leukemic stem cells (LSCs) was demonstrated. In AML, it has been proposed that LSCs propagate the disease and are able to replenish the leukemic bulk after complete remission suggesting that LSC may be endowed with drug resistance properties. However, whether such properties are due to extrinsic or intrinsic molecular mechanisms, fully or partially supported by molecular crosstalk between LSCs and surrounding BM micro-environment is still matter of debate. In this review, we focus on adhesion molecules that have been involved in HSCs or LSCs anchoring to BM niches and discuss if inhibition of such mechanism may represent new therapeutic avenues to eradicate LSCs.

## Introduction

**Figure 1:**
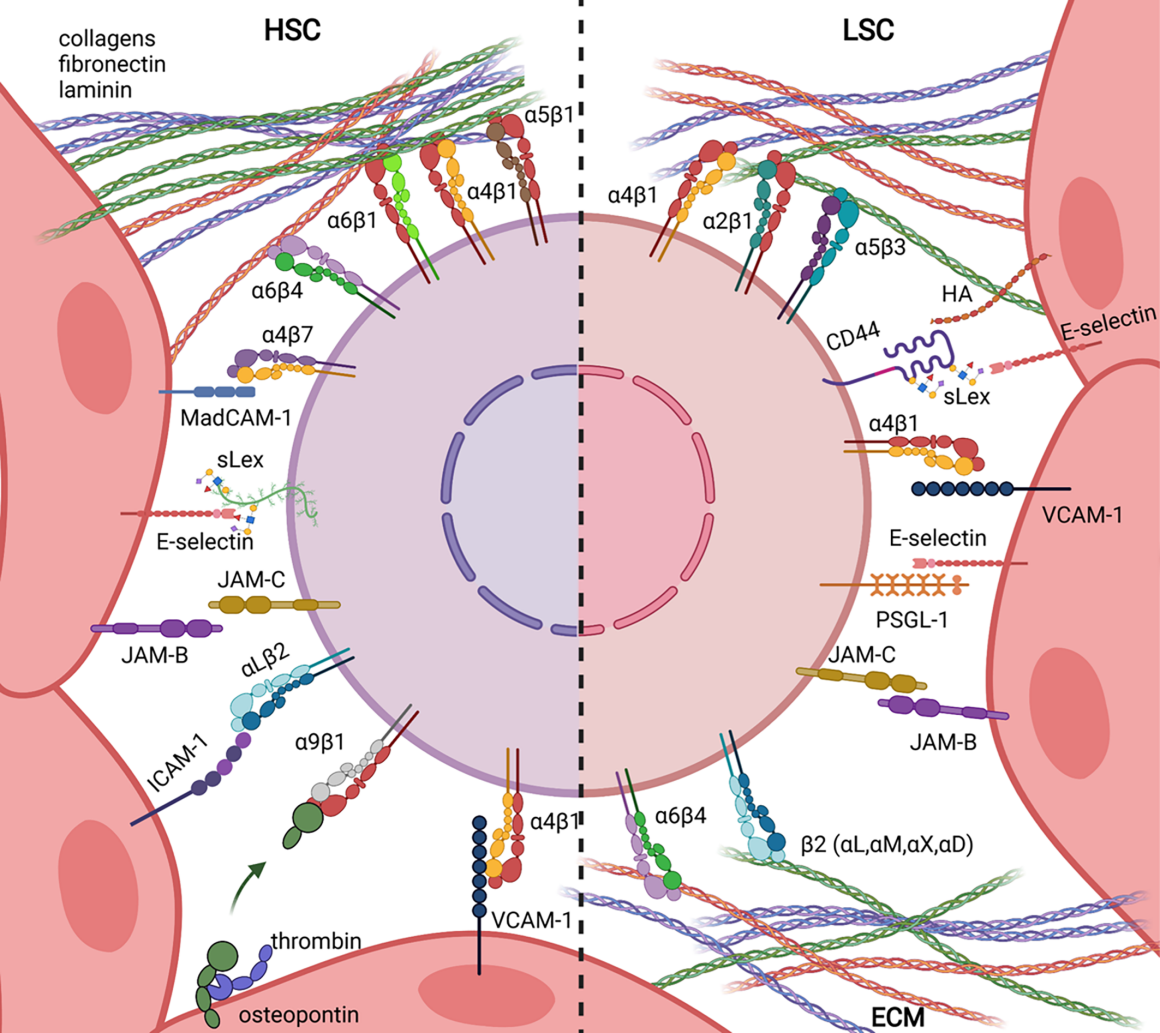
Ligand/Receptor adhesion pairs involved in Haematopoietic Stem Cell (HSC, left) and Leukemic Stem Cell (LSC, right) retention in bone marrow niches.

Haematopoiesis takes place in the bone marrow of adult mammals and is the process leading to the formation of blood components throughout life. Haematopoietic stem cells (HSCs) are at the apex of the haematopoietic hierarchy and are able to self-renew and to differentiate into all blood cell types. The balance between differentiation and self-renewal is controlled by intrinsic properties of HSC and extrinsic cues delivered by the bone marrow microenvironment in micro-anatomical sites called “niches”. The concept of niche has been formulated by R. Schofield in 1978 who proposed that stem cell association with other cells prevents maturation while its progeny proliferate and differentiate, unless they can occupy a similar ‘niche’ (1). Although this working hypothesis turned to be true, its formal proof has long time been hampered by the lack of methods allowing precise localization of un-manipulated HSC within their niche (2, 3). In addition, because HSC activity has been essentially studied in transplantation assays, it has been difficult to decipher whether experimental assays were measuring intrinsic HSC stemness of engrafted cells or their ability to find a supportive niche in which they can self-renew (4, 5). The development of constitutive knock-out mouse models in the early 90’s, and conditional or inducible models later on, has represented a breakthrough to study the contribution of niche components to mammalian haematopoiesis (6, 7). Accordingly, a bibliographic search using combination of the words “haematopoiesis, adhesion and niche” reveals that only seven publications combine such words between 1989 and 2000, while more than hundred papers have been published thereafter. This likely indicates that adhesion was initially considered as an intrinsic property of HSC, while it has been integrated to the niche concept later on. This review is focused on adhesion molecules implicated in HSC or acute myeloid LSC interaction with the BM microenvironment (**Figure 1**).

## Adhesion Molecules Involved in HSC Retention in the Bone Marrow

With the exception of CD44, haematopoietic adhesion molecules belong to the immunoglobulin superfamily (Ig Sf), the cadherin family, the selectin family or the integrin family. Adhesion molecules promote cell/cell or cell/extracellular-matrix (ECM) interactions and deliver survival signals to haematopoietic cells. Reciprocally, stromal and endothelial cells express adhesion molecules interacting with haematopoietic cells or ECM contributing to the maintenance of bone marrow architecture.

### Integrins

Integrins are non-covalent heterodimers of α and β chains. In mammals, 18 α and 8 β subunits form 24 different integrin heterodimers involved in embryonic development and maintenance of tissue homeostasis. α/β chain pairing and integrin interaction with ECM, cell surface molecules or soluble factors have been extensively reviewed in the past and will not be described in further details here (8–11).

**Figure 2:**
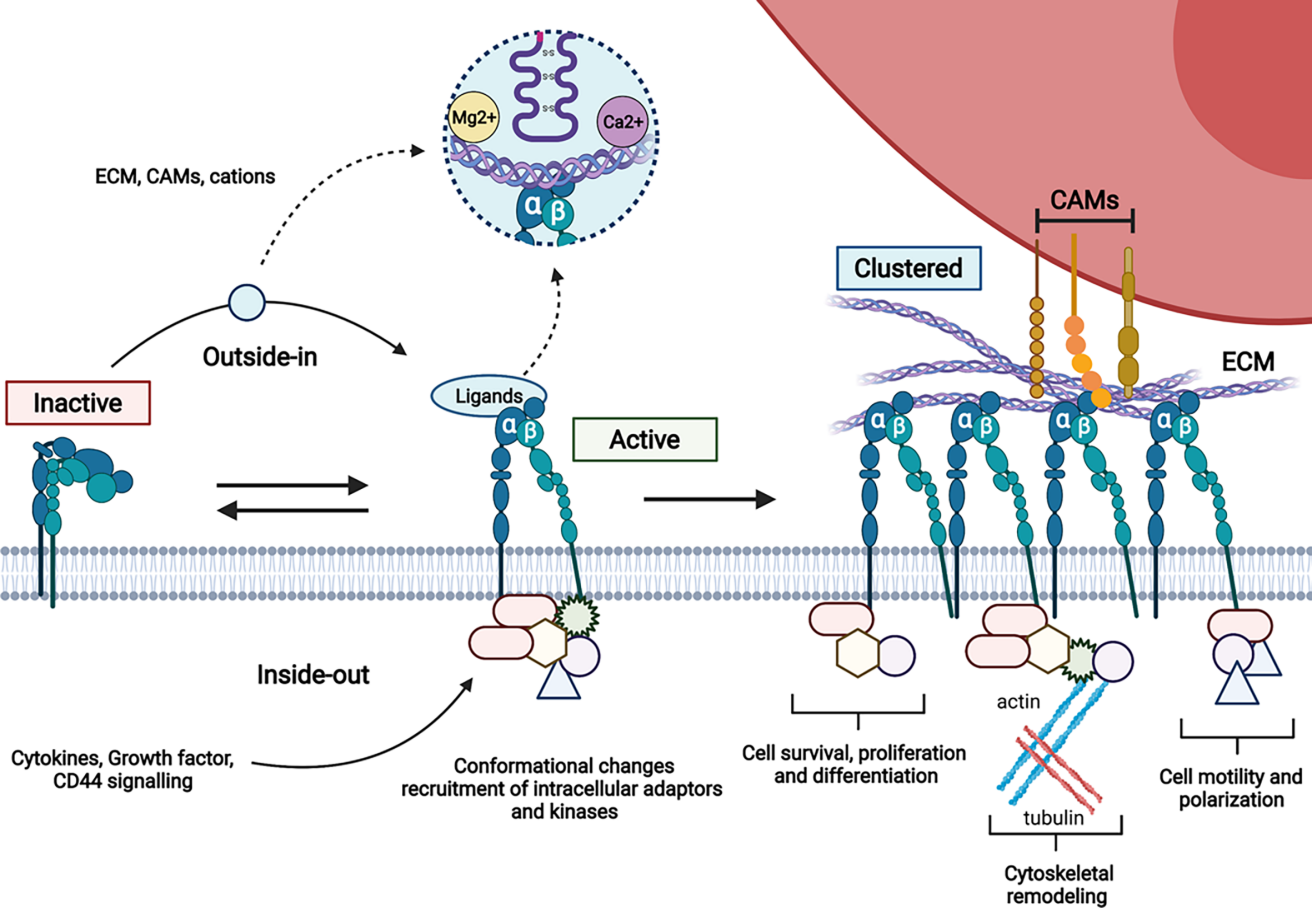
Schematic representation of integrin activation. The variety of intracellular protein complexes involved in integrin signalling (kinases, adaptors…) is depicted by forms recruited to the cytoplasmic tails of integrins.

One key property of integrins is that they can be expressed in inactive, activated or clustered state on the surface. The switch between inactive and active state results in increased ligand affinity as a consequence of inside-out or outside-in signalling. Integrin clustering further induces cytoskeleton rearrangement and enhanced cell signalling (**Figure 2**).

Among α_4_β_1_, α_5_β_1_, α_6_β_1_, α_6_β_4_and α_9_β_1_ integrins that have been involved in interaction of HSC with bone marrow microenvironment (12–18), α_4_β_1_ is the most studied. The integrins α_4_β_1_ and α_5_β_1_ are activated by inside-out signalling that involves cytokines and divalent cations present in the bone marrow microenvironment, suggesting that they are essential for HSC retention in the bone marrow (19, 20). Accordingly, HSPC mobilization using G-CSF is correlated to decreased α_4_ integrin expression (21) and deletion or inhibition of α_4_β_1_ integrin result in accumulation of HSC in the blood circulation (22–25). Similar results were obtained using antibody against VCAM-1, suggesting a central role of α_4_β_1_/VCAM-1 axis in HSC retention in the bone marrow (26). This is consistent with the finding that β_1_ null HSC fail to engraft in irradiated recipient and that β_1_ null HSC from chimeric embryos are unable to seed foetal liver (27, 28).

Along this line, β_7_-deficient mice do not have defects in HSCs function (29), while interaction between α_4_β_7_ and MadCAM-1 (mucosal addressin cell adhesion molecule-1) accounts for half of the α_4_-integrin mediated homing activity to the bone marrow (30, 31). Therefore, it seems that β_1_ integrin heterodimers play a prominent role in bone marrow HSC retention as further supported by the fact that the dual α_9_β_1_/ α_4_β_1_ inhibitor BOP ((N-(benzenesulfonyl)-L-prolyl-L-O-(1-pyrrolidinylcarbonyl)tyrosine) induces a rapid mobilization of HSCs including those that are located in the endosteal region which bind thrombin-cleaved osteopontin with high affinity (32). This is also supported by the finding that patients treated with natalizumab, an anti-α_4_ integrin antibody, present increased levels of circulating CD34^+^ progenitor cells associated with an higher migratory profile as compared to GM-CSF mobilization (33, 34).

Finally, it has recently been reported in zebrafish that VCAM-1^+^ patrolling macrophages can interact with HSCs in an α_4_β_1_ dependent manner and contribute to their retention in the niche (35). This study confirms earlier findings in mouse models showing that macrophages contribute to HSC retention within niches through integrin-mediated interactions (36–38).

### Selectins

The selectin family encompasses three members: E- (Endothelial), P- (Platelets) and L- (Leukocyte) selectins expressed by endothelial cells (E- and P- selectins), platelets (P-Selectin) and leukocytes (L-Selectin). They have been initially involved in the rolling of haematopoietic cells along vessels in flowing blood (39–41).

The minimal requirements for Ca^2+-^dependent ligand binding to selectins are the tetra-saccharides Sialyl Lewis X (Sle^x^) and Sialyl Lewis A (Sle^A^) (42, 43). As reviewed elsewhere (44), Sle^x^ and Sle^A^ synthesis requires several enzymes including α (1–3)-fucosyltransferase activities as illustrated by defective selectin-dependent leukocyte trafficking in FucT-VII deficient mice (45). This is reminiscent of the phenotype of P-Selectin deficient mice that harbour elevated number of circulating neutrophils, loss of leukocyte rolling in mesenteric venules and delayed leukocyte recruitment in peritonitis model (46). In contrast, E-selectin deficient mice have no defect in neutrophils trafficking suggesting a compensatory mechanism mediated by P-selectin (47).

The study of double knockout mice for E- and P-selectin has revealed defect in haematopoiesis with increased extramedullary erythropoiesis and reduced haematopoietic progenitor cell homing in irradiated deficient mice upon transplantation (41, 48). However, such functions were mostly attributed to HSPC homing and it is only in 2012 that E-selectin was shown to mediate HSC proliferation at the expense of self-renewal (49). In contrast to E- and P- Selectin, early haematopoietic defects in L-Selectin-deficient mice have not been reported so far (50).

### Cadherins

Cadherins are transmembrane glycoproteins characterized by tandemly repeated sequence motifs in their extracellular segments that allow homophilic interactions in a Ca^2+^ dependent manner (51). N-cadherin is not only expressed by neural cells but also by HSCs and spindle shaped osteoblastic cells lining the bones, called “Spindle-shaped N-cadherin^+^CD45^–^ Osteoblastic” (SNO) in the original publication. Because conditional inactivation of BMP receptor type IA (BMPRIA) led to expansion of both SNO and HSC, with asymmetric N-Cadherin distribution between SNO and HSC adjacent cells, it has been proposed that N-cadherin-mediated adhesion contributes to HSCs maintenance in endosteal niche (52). This concept was further supported by the fact that the knock-out of N-cadherin in LSK cells impairs long term engraftment in the bone marrow but not in the spleen (53). However, the latter demonstration used LSK cells, a compartment in which less than 20% of the cells are HSCs. Therefore, the function of N-cadherin mediated adhesion in HSC maintenance has been challenged in several studies. First, it was demonstrated that N-cadherin is not expressed on purified HSCs and that osteoblasts are dispensable for HSC maintenance (54). Second, the conditional deletion of N-cadherin in HSC using Mx1-Cre did not affect haematopoiesis, nor did its specific deletion in osteoblasts (55–57). Therefore, the controversial function of N-cadherin in HSC maintenance has been revisited in the light of the methodology used to study its function (engraftment *versus* knock-out) and with respect to heterogeneous expression of N-cadherin by HSC subsets (58, 59). This led to the most recent concept that N-cadherin mediated adhesion of HSC to BM stromal progenitor cells (BMSPC) may only be revealed during emergency haematopoiesis such as the one needed by “reserve” HSC to survive chemotherapy (60).

### Ig Sf Adhesion Molecules

Several Ig Sf adhesion molecules such as ALCAM (CD166), ESAM, JAM-A or JAM-C are expressed by HSPCs and BM stromal or endothelial cells (61–64). Some others such as ICAM-1 or VCAM-1 are expressed in the BM microenvironment and interact with integrins expressed by HSPCs or contribute to more complex adhesive networks involving IgSf/Integrin as well as IgSf/IgSf interactions such as the JAM family members (65–68). Therefore, early haematopoietic defects reported for IgSf deficient animals have to be interpreted with caution unless specific conditional knock-out mouse models are combined with orthogonal methods such as long-term engraftment. Defects in early haematopoiesis following knockout have been reported for ALCAM, ESAM, VCAM-1, JAM-C, JAM-B and ICAM-1 (**Table 1**).

**Table 1:** Knock-out mice of Ig Sf molecules presenting haematopoietic defects.

Adhesion molecule	Year	Ligands	Altered phenotype	Haematopoietic phenotype	References
**ICAM-1**	1994	α_L_β_2_	cardiovascular, cellular, digestive/alimentary, growth/size/body, haematopoietic, homeostasis, immune, mortality/aging, neoplasm, vision/eye	Expansion of Lt-HSC compartment associated with impaired quiescence and myeloid expansion	(69, 70)
**VCAM-1**	1995	α_4_β_1_	cardiovascular, embryo, growth/size/body, homeostasis, mortality/aging, haematopoietic	Increased frequencies of circulating progenitors	(65, 71)
		α_4_β_7_			
**ESAM**	2003	ESAM	cardiovascular, cellular, growth/size/body, haematopoietic, immune	Increased HSCs frequency and proliferation compared to wild-type mice	(63, 72)
**ALCAM (CD166)**	2004	ALCAM	nervous system, vision/eye, haematopoietic	Defects in Lt-HSC engraftment although no differences in absolute numbers of HSCs were observed	(61, 73, 74)
		CD6			
**JAM-C**	2004	JAM-C	behaviour, cardiovascular, cellular, craniofacial, digestive/alimentary, endocrine/exocrine, growth/size/body, haematopoietic, immune, integument, mortality/aging, nervous system, reproductive, respiratory, skeleton	Increased number of CMPs	(75–77)
		JAM-B			
		α_M_β_2_			
**JAM-B**	2011	JAM-C	haematopoietic, homeostasis, mortality/aging, skeleton	Loss of quiescent HSCs and exacerbated response to mobilizing agent	(78)
		α_4_β_1_			

## Adhesion Molecules Involved in LSC Retention in the Bone Marrow

Similar to HSCs, LSCs are retained into specialized microanatomical sites by adhesive interactions. Indeed, AML development originates from LSC which share with HSCs the ability to self-renew (79, 80). After disease initiation, acute myeloid leukemic burst is accompanied by a remodelling of bone marrow niches that alters the physiological adhesive network of HSC (81–83). Whether adhesive remodelling occurs already at disease initiation in immunocompetent context remains to be addressed, but several adhesive Ligand/Receptor pairs have been involved in AML development in mouse models. Among them, only a limited number of Ligand/Receptor pairs that cross barrier species have been validated as putative therapeutic targets in preclinical setting using patient derived xenograft (PDX) models. This has encouraged some clinical trials targeting LSC adhesion to the niche in order to sensitize these cells to chemotherapy as recently reviewed by A. Villatoro et al. (84). In the next section, we will discuss the adhesion molecules known to contribute to LSC stemness maintenance that belong to the emerging class of adjuvant therapies for LSC eradication in AML.

### CD44

CD44 is a class I transmembrane glycoprotein that does not belong to an adhesion molecular family and that interacts with ECM ligands such a as osteopontin, fibronectin or hyaluronan (HA). When CD44 is sialo-fucosylated and bears Sle^X^ glycan, it is called HCELL and interacts with E- and L-selectin (85, 86). In addition, several isoforms of CD44 are generated by alternative splicing and associated with different cellular processes (87). CD44 isoforms are widely expressed on AML cells and expression of the CD44-6v isoform has been associated with poor prognosis (88, 89). Functionally, CD44 has been involved in AML cell adhesion to bone marrow stromal cells (90, 91) and ligation of CD44 with HA or activating antibodies such as H90 has been shown to reverse differentiation blockage in AML cells (92). The same H90 activating antibody inhibited homing of AML-LSC to microenvironmental niches reducing the leukemic burden in a PDX setting. This was attributed to opposing effects of the H90 antibody which increases adhesion of normal CD34^+^CD38^-^ cells to HA but inhibits adhesion of CD34^+^CD38^-^ AML blasts to HA (93).

### Integrins

Overexpression of the integrins α_M_β_2_ (CD11b/Mac1), α_2,_ α_6_ and α_4_β_1_ by AML cells has been associated with poor prognosis (94–96). Indeed, it has early been shown that both β_1_ and β_2_ integrin chains are necessary for AML blast adhesion to BM stromal cells (97).

Among the β_1_ integrins, α_4_β_1_ seems to play the most prominent role through its interaction with fibronectin (FN) and VCAM-1. Interaction of integrin α_4_β_1_ with FN protects AML cells from chemotherapy and is associated with the maintenance of minimal residual disease (MRD). Treatment with a blocking antibody against α_4_β_1_ abrogates chemoresistance and MRD in mice (98). Similarly, integrin α_4_β_1_ interaction with VCAM-1 contributes to drug resistance by activating NF-κB pathway in BM stromal cells which is essential to promote chemoresistance in leukemic cells as demonstrated by inhibition of NF-kB signalling (99). This study illustrates the reciprocal crosstalk between LSC and stromal cells since NF-κB activation in stromal cells upregulates VCAM-1 which serves as a positive feedback loop for leukemic cell adhesion to stromal cells.

More recently, the interaction between the integrin α_2_β_1_ and collagen has been shown to confer doxorubicin chemoresistance *via* the inhibition of Rac-1 (100). This protective effect is reversed by anti-α_2_β_1_. Although these studies show the therapeutic potential of integrin inhibition in AML, they do not formally prove that LSC are more addicted to integrin-mediated adhesion than normal HSC. To find such differential adhesive cues, Ebert and collaborators have used results from pooled *in vivo* shRNA screens. They have found that the integrin α_v_β_3_ is essential for leukemic initiation and maintenance but dispensable for normal HSPC activity (101). This was attributed to constitutive activation of Syk, a candidate therapeutic target in AML, that is phosphorylated upon engagement of surface receptors including not only α_v_β_3_ integrin, but also β_2_ integrins (102, 103). In summary, integrin signalling converging toward specific activation pathway such as NF-kB or Syk may represent attractive therapeutic targets.

### E-Selectin

E- and P-Selectins are constitutively expressed by bone marrow endothelial cells and play a role in HSPC rolling on micro vessels (39, 104, 105). However, they induce contrasting effects in HSPC upon interaction *in vitro* (86, 106–108). The study of early haematopoiesis in E-Selectin deficient mice (*Sele* ^-/-^) has revealed that inhibition of E-Selectin *in vivo* increases dormancy and self-renewal of HSC (49). This is not mediated by the conventional ligands of E-Selectin since HSC isolated from mice deficient for P-selectin glycoprotein ligand-1 (Psgl-1 encoded by *Selplg*), HCELL (*Cd44*) or both do not present increased dormancy. In contrast, LSC of AML make a different selectin receptor usage that promotes AML cell survival. Indeed, leukemic cells present alterations in glycosylation which leads to expression of fucosylated ligands such as PSGL-1 (CD162) that activate PI3K/Akt survival pathway (109, 110). Even more interesting is the fact that inhibition of E-selectin interaction with its ligands using a glycomimetic stimulates proliferation of AML blast while dampening HSC cycling. Since these finding have been confirmed in preclinical mouse models, this led to the opening of phase II/III clinical trials combining inhibition of E-selectin with conventional chemotherapy in AML (NCT03616470, NCT03701308).

### Ig Sf Adhesion Molecules

Most of the Ig Sf molecules expressed by normal HSC are also expressed by LSCs in AML, however only few of them allows enrichment of cells with leukemic initiating activity associated to poor prognosis. We have shown that JAM-C is expressed by a fraction of LSCs presenting high activation of Src kinase family and enriched for leukaemia initiating activity. Increased frequencies of JAM-C expressing cells identify AML patients with poor disease outcome (111). This has been confirmed in an independent study on a large cohort of AML patients (112, 113). The “CD34^+^ CD38^low^ CD123^+^ CD41^-^ JAM-C^+^” cells are enriched tenfold for LSCs as compared to cells lacking JAM-C expression within the same compartment suggesting that JAM-C may play a cell-autonomous signalling function at the transition between healthy HSC and LSC. This would be consistent with results showing that PDX or AML cell line engraftment of JAM-C-expressing cells is only partially dependent on JAM-B expression by recipient mice and with results showing that silencing JAM-C expression is sufficient to decrease Src family kinase activation (111). This could be due to promiscuous cis-interactions between JAM-C and the integrin α_4_β_1_ since JAM-B has been shown to bind α_4_β_1_ when interaction is facilitated by the simultaneous engagement with JAM-C (67).

NCAM1(CD56) is another Ig Sf molecules whose expression is correlated with poor overall survival in AML with t(8;21) (q22; q22) and highly expressed by LSC in mouse AML models using MLL-AF9 or Hoxa9-Meis1 as driver translocations (114). NCAM1 expression confers drug resistance to AML cells and knockdown of NCAM1 sensitizes blasts to genotoxic agents (115). This is likely due to constitutive activation of the MEK-ERK pathway, similar to what has been reported during neural development (116). These two examples pave the way for the use of Ig Sf molecule expression to stratify patients eligible to treatments targeting downstream signalling pathways such as Src or Mek/Erk.

## Outlook

Recent studies have shown that HSC niches are altered during AML development with strong coordinated changes of the osteolineage and endothelial compartments, and alterations of the mesenchymal compartment occurring early during leukemic development. Whether such alterations depend on adhesive interaction of leukemic initiating cells with BM microenvironment resulting in localization of LSCs in specific sites remain to be defined, but it seems that LSC take advantage of pre-existing adhesive pathways in the niche to maintain survival signals and dormancy that protect them from chemotherapies. Therefore, the selective disruption of LSC from their niche by targeting single adhesion molecule remains a major limitation for current therapies. A better knowledge of the differences between LSC/Niche and HSC/Niche integrated adhesive networks will help refining specificity of therapeutic strategies directed against adhesive cues.

## Author Contributions

JG wrote and revised the manuscript. CT, CF, and SM revised the manuscript and MA-L supervised the work. All authors contributed to the article and approved the submitted version.

## Funding

JG was supported by a grant from the French Society of Haematology (SFH). CT was supported by a grant from the ligue nationale contre le cancer. The work was supported by the Ligue Nationale Contre le Cancer, ELN2020.

## Conflict of Interest

The authors declare that the research was conducted in the absence of any commercial or financial relationships that could be construed as a potential conflict of interest.

## Publisher’s Note

All claims expressed in this article are solely those of the authors and do not necessarily represent those of their affiliated organizations, or those of the publisher, the editors and the reviewers. Any product that may be evaluated in this article, or claim that may be made by its manufacturer, is not guaranteed or endorsed by the publisher.
